# The Effect of Citrus Aurantium Aroma on the Sleep Quality in Postmenopausal Women: A Randomized Controlled Trial

**DOI:** 10.30476/IJCBNM.2021.90322.1693

**Published:** 2022-04

**Authors:** Zahra Abbaspoor, Amir Siahposh, Nahid Javadifar, Shahla Faal Siahkal, Zeynab Mohaghegh, Foruzan Sharifipour

**Affiliations:** 1 Department of Midwifery, Menopause Andropause Research Center, School of Nursing and Midwifery, Ahvaz Jundishapur University of Medical Sciences, Ahvaz, Iran; 2 Department of Pharmacology, Medical Plants Research Center., School of Pharmacy, Jundishapur University of Medical Sciences, Ahvaz, Iran; 3 Department of Midwifery, School of Nursing and Midwifery, Ahvaz Jundishapur University of Medical Sciences, Ahvaz, Iran

**Keywords:** Aromatherapy, Citrus aurantium, Postmenopausal women, Sleep quality

## Abstract

**Background:**

One of the most common problems in menopausal women is sleep disturbance. Citrus aurantium has sedative, hypnotic, and anti-anxiety effects. The aim of this study was to investigate the effect of Citrus aurantium aroma on the sleep quality of postmenopausal women.

**Methods:**

This was a double-blind randomized controlled trial that was conducted from Feb to Dec 2019 on 80 postmenopausal women who suffered from sleep disturbances. The participants were assigned into two groups randomly. Women in the intervention group were requested to use 2 drops of essential oil of Citrus aurantium, twice a day, for 4 consecutive days in a week, for 4 weeks as inhalation. The control group received almond oil in the same way. The quality of sleep was evaluated using the Pittsburgh Sleep Quality Index (PSQI) before the intervention and 4 weeks after the intervention started. The data were analyzed using the SPSS statistical software, version 21, and P<0.05 was considered statistically significant.

**Results:**

After 4-weeks of intervention, the mean score of sleep quality was significantly lower in the Citrus aurantium group compared to the control group (5.75±1.33 vs 13±1.59, P<0.001). In the intervention group, all dimensions of PSQI were improved significantly (P<0.001).

**Conclusion:**

The results of this study showed that the aroma of Citrus aurantium essence could significantly improve the sleep quality of postmenopausal women. Therefore, it is recommended that health care providers should inform the postmenopausal women and advise them to use this intervention for reduction of sleep disorders.

**Trial Registration Number:**

IRCT20160427027633N7

## INTRODUCTION

Menopause is considered as one of the most important periods of women’s life. ^1^ Because of the increase in life expectancy, women are expected to live longer after menopause. ^2 , 3^ Given the rising number of postmenopausal women throughout the world, the population of menopausal women is predicted to reach 1.1 billion by the year 2025 around the globe, and the figure will be nearly 5 million in Iran in 2021. ^4^

Due to lack of sexual hormones in the body after menopause, women experience many changes such as hot flashes, palpitations, depression, anxiety, sleep disorders, sexual problems, myalgia, and cardiovascular diseases. ^5^ Sleep disorder is one of the most common complaints in menopausal women, and menopause is often considered as a starting or intensifying point for sleep problems. ^6^ The prevalence of this disorder has been reported 40.4% among Iranian postmenopausal women. ^7^

Poor sleep quality is associated with negative health sequela. ^8^ Sleep disorders also interfere with an individual’s performance and productivity, memory, cognitive functions, and social interaction. ^9^

Hormonal changes in the menopausal period can contribute to sleep disorders. ^10^ Particularly, low levels of estrogen cause a reduction in melatonin and serotonin levels, which are essential for sleep. ^11^ Hot flushes have also been reported to be associated with poor sleep quality. ^12^

Hormonal therapy, sedative and hypnotic drugs are the treatment methods for sleep disorders, but they can cause harmful effects such as breast cancer, thrombosis, drowsiness, and impaired mental ability. ^13 , 14^

In contrast, there are various complementary therapies including mind–body practices, acupuncture, cognitive behavioral therapy, and music therapy used for solving sleep problems which are less costly and have minor or no side effects. ^15^ Over the past decade, there has been a significant increase in the use of complementary and alternative therapies in treatment of the menopausal symptoms such as sleep disorders, among which aromatherapy is relatively new and deserves more scholarly attention. ^16 , 17^

Aromatherapy is the use of essential oils extracted from aromatic plants for therapeutic purposes, and it affects the brain, mind, and body. ^9^ Citrus aurantrium essence, known as neroli oil, belongs to the rutaceae family and contains alkaloid, linalool, linanil acetate, msyrine, limonene, limonoids, and flavonoids. ^18^ It has sedative, hypnotic, and anti-anxiety effects. ^19 - 21^ The linalool in the Citrus aurantrium has been reported to exert sedative and hypnotic effects. ^20^

Aromatherapy studies that have focused on menopausal symptoms have shown that aromatherapy has an alleviating effect. ^17 , 22^ According to one study, aromatherapy with citrus aurantium improved the sleep quality in patients with type 2 diabetes. ^23^ In another study, it was found that the aroma of citrus aurantium improved the quality of life of women after menopause. ^24^ Previous studies have checked and proved the effectiveness of citrus aurantium in reducing pain and anxiety in different patient populations. ^21 , 25^

Given the growing population of postmenopausal women and the high prevalence of sleep disorders among them, it is necessary to use non-pharmacological interventions to manage sleep problems due to their low side effects. To the best of our knowledge, no study has yet assessed the effects of Citrus aurantium aroma on the quality of sleep in postmenopausal women. Therefore, this study was conducted to investigate the impact of aromatherapy using Citrus aurantium on postmenopausal women’s sleep quality.

## MATERIALS AND METHODS

This randomized, controlled trial was conducted on 80 postmenopausal women who suffered from sleep disturbances and referred to two health centers in Ahvaz, Iran from Feb to Dec 2019. 

The sample size was calculated for each group based on the results of a previous similar study. ^23^ The following equation was used to calculate the sample size. 

$N=\frac{{\left(Z_{1-\frac{\alpha }{2}}+Z_{1-\beta }\right)}^{2}\times (S_{1}^{2}+S_{2}^{2})}{{\left(\mu_{1}-\mu_{2}\right)}^{2}}$

In this equation, α=0.05, β=0.1, μ_1_=4, μ_2_=0.286, S_1_=5, and S_2_=3.72, and n=30. We added 25% for attrition, and the total number of individuals in each sample group was calculated to be 40.

The inclusion criteria were age of 45-60 years, menstrual cessation for at least 12 months, ability to read and write, the score ≥5 from the Pittsburgh sleep quality questionnaire, no mental and physical diseases, no smoking, no drinking alcohol, no history of allergic rhinitis or a known respiratory problem such as asthma, no experience of stressful events (divorce, death of first-degree family members, etc.) during the 6 months prior to the study, and available health records in the health care center. The exclusion criteria were being unwilling to continue participation, and not completing the intervention course for any reasons. 

After receiving approval from the Ethics Committee of Ahvaz Jundishapur University of Medical Sciences, the researcher referred to two health centers for data collection. Eligible postmenopausal women were randomly assigned into intervention (n=40) and control (n=40) groups. The allocation sequence was determined by computer using a table of random numbers with a block size of 6 and a 1:1 allocation ratio. The Citrus aurantium and almond oil were kept in identical containers and coded by a pharmacist. For blinding the allocation, one unique code was allocated to each woman, and all codes were kept in an opaque envelope until the time of intervention. Neither the statistician (the one who did the data analysis) nor the researcher (the one who was involved in sampling) was aware of group alocations.

Women in the intervention and control groups were requested to put 2 drops of essential oil of Citrus aurantium 10% or aroma of almond oil on their (left or right) forearm skin to receive it as inhalation, twice a day (10 a.m. and 10 p.m.), for 4 consecutive days per week, and continued it for 4 weeks. Each woman was instructed to be in a comfortable position and place her forearm 30 cm away from her nose and inhale the fragrance for five minutes with normal breathing as reported by Malakuty et al. ^26^ One of the researchers made phone calls to the participants to make sure of the correct usage and check for any adverse effect. Before the start of the intervention and at the end of the fourth week after the start of the intervention, the participants were asked to return to the center to fill out the questionnaire of Pittsburgh Sleep Quality Index (PSQI) again.

Essential oil of Citrus aurantium 10% was purchased from a pharmaceutical research center in Tehran (Iran) and approved by the Faculty of Pharmacy of Ahvaz Jundishapur University of Medical Sciences. Odorless almond oil, diluted with propylene glycol was purchased from a medicinal herbs market in the city of Kermanshah (Iran). Based on previous studies, almond oil was used in the control group. ^22 , 27^ The concentration of essential oil was 10%, namely 10 mg of Citrus aurantium essential oil in 100 ml of odorless almond oil and diluted with propylene glycol. Propylene glycol maintained the stability of the essential oil. The amount of essential oil required for a 4-week intervention was provided to the participants at the beginning of the study and stored in the droppers.

The data collection instruments included a socio-demographic information form and the PSQI questionnaire. The socio-demographic information form was used for recording data such as age, education level, occupation, age of menopause, body mass index, marital status, medication use, and number of children.

The PSQI consists of 9 questions in 7 domains (mental quality of sleep, sleep latency, sleep duration, adequacy of sleep, sleep disturbances, use of sleeping pills, and daily dysfunction). Sum of the score of the 7 domains is the total score of the questionnaire, which ranges from 0 to 21. A score ≥ of 5 indicates that the person has a sleep problem. 

For measuring reliability, internal homogeneity of the PSQI scale was measured by Cronbach’s alpha coefficient and the seven components of the scale had a Cronbach’s α of 0.83. For analysis of validity, ANCOVA test was used to compare the patient groups for PSQI global and component scores, and the control subjects differed from all patient groups. Also, the validity of PSQI was determined by comparing PSQI estimates of sleep variables with those obtained by Polysomnography. The result of t-test showed no differences between the PSQI estimates and laboratory findings for sleep latency, but PSQI estimates of the past month’s usual sleep duration and effeiciency were greater than those obtained during polysomnography (t=9.98 and 4.50, respectively; both P<0.001). A global PSQI score more than 5 showed a diagnostic sensitivity of 89.6% and specificity of 86.5% in distinguishing good and poor sleepers. ^28^

In Iran, Farahi et al. evaluated the psychometric properties of the Persian version of PSQI and Cronbach’s alpha coefficient for all subjects was 0.77, indicating a satisfactory internal consistency (0.72-0.78). ^29^ The corrected component–total correlations of six out of seven PSQI subcomponents were above the minimum acceptable level of 0.4, and the correlation coefficient of “sleep duration” was slightly lower than 0.4 (i.e., 0.30). ^30^ The concurrent validity of the PSQI is further supported by its modest correlation with the GHQ-12 (general health questionnaire-12) scores (r=0.252, P<0.001). ^31^ Also, the receiver operating characteristic (ROC) analysis was used to evaluate the criterion validity. According to ROC analysis, using a PSQI cutoff value of 6 resulted in high sensitivity (93.6%) and high specificity (72.2%) for the discrimination of insomniac patients from the control group. The area under the ROC curve was 0.92 (P<0.001). Overall, the Persian version of the PSQI received optimal psychometric properties for assessment. ^32^

All data were entered into SPSS version 23. Analyses were conducted adopting an intention – to - treat (ITT) approach. Missing values were imputed by last observation procedure. The normal distribution of data was assessed using the Kolmogorov-Smirnov test. For assessing the differences between the two groups, independent t-test or Mann-Whitney U test was used. The paired t-test or its non-parametric equivalent (Wilcoxon test) was used to evaluate within-group differences. P values less than 0.05 were considered statistically significant.

The Ethics Committee of Ahvaz Jundishapur University of Medical Sciences, Ahvaz, Iran (code: IR.AJUMS.REC.1398.162) approved this study. All participants were fully aware of the study objectives and procedures and signed written informed consent. They were assured that their information would remain confidential. They were also informed that they could leave the study at any stage of the research. 

## RESULTS

**Figure 1:**
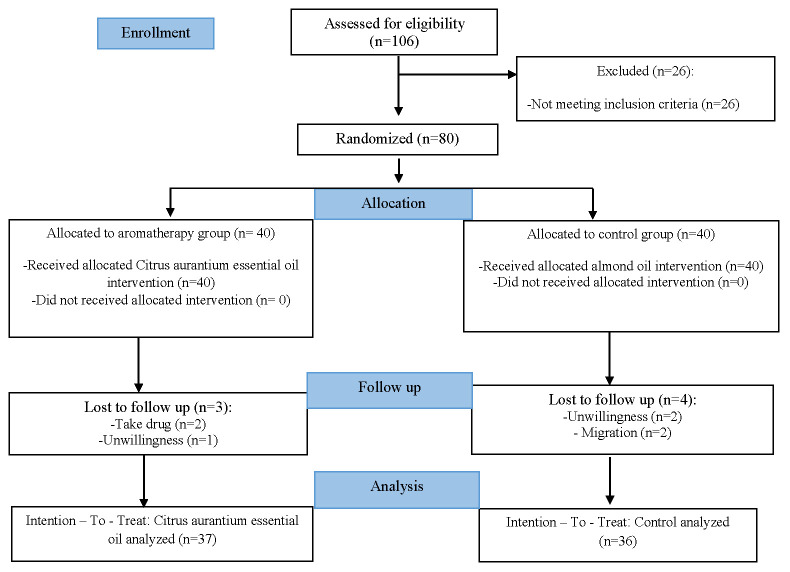
Flow diagram of the recruitment and retention of participants in the study

A total of 80 women were initially enrolled in this study. However, 3 women in the Citrus aurantium group and 4 in control group withdrew from the study. The reasons for drop-out are listed in Figure 1. 

Table 1 shows the socio-demographic characteristics of the participants in the two groups of Citrus aurantium and almond oil. The participants’ mean age was 59.2±5.94 years, and their mean menopausal age was 52.03±2.73 years. No significant differences were found between the groups in terms of descriptive characteristics (P>0.05).

**Table 1:** Socio-demographic characteristics of the Citrus aurantium and control groups

Variables		Citrus aurantium	Almond oil	P value
		n=40	n=40	
		Mean±SD	Mean±SD	
		Or N (%)	Or N (%)	
Age (years)		59.69±5.18	58.72±5.91	*0.46
Age of menopause		52.50±2.84	51.56±2.63	*0.15
Body Mass Index		29.11±3.58	29.57±3.79	*0.59
Education level	Primary high school	19(47.5)	15(37.5)	**0.30
	Secondary high school(diploma)	15(37.5)	15(37.5)	
	University education	6(15.0)	10(25.0)	
Occupation	Employed	6(15.0)	7(17.5)	**0.71
	Retired	17(42.5)	13(32.5)	
	House wife	14(35.0)	15(37.5)	
	Other	3(7.5)	5(12.5)	
Marital status	Single	6(15.0)	4(10.0)	**0.62
	Married/common-law	26(65.0)	29(72.5)	
	Divorced/separated	8(20.0)	7(17.5)	
Number of children	≤2	8(20.0)	8(20.0)	**0.98
	2-4	22(55.0)	21(52.5)	
	>4	10(25.0)	11(27.5)	
Medication used	Hormone therapy only	0 (00.0)	2(05.0)	**0.52
	Psychoactive medication only	14(35.0)	13(32.5)	
	Hormone therapy, psychoactive medication	2(05.0)	3(07.5)	
	No medication	24(60.0)	22(55.0)	

*Independent t-test; **chi-square test

Table 2 shows the mean of PSQI scores in the group who received Citrus aurantium and the almond oil group at the baseline (12.08±2.1vs. 12.4±1.65, respectively) (P=0.46). The mean±SD of PSQI scores in the group who received Citrus aurantium were significantly lower than those of the control group at the end of the trial (5.75±1.33 vs. 13.0±1.59, respectively) (P<0.001). Based on the paired t-test, there was no significant difference between the baseline and the end of the intervention in the control group (P=0.07), whereas there was a significant difference at the baseline and end of the intervention in the Citrus aurantium group (P<0.001). According to the between-group analysis, women who received Citrus aurantium aroma had a signiﬁcantly greater improvement in the scores of the seven domains of PSQI compared with those who used almond oil (P<0.001). Within-group analysis showed no significant differences in the mean scores of the seven domains of PSQI in the control (P>0.05) group before and after the intervention. However, a significant difference was observed in the intervention group in this regard before and after the intervention (P<0.001). No adverse effects such as headache, dizziness, dyspnea, nausea and vomit were reported following inhalation of the aroma of Citrus aurantium and almond oil during the intervention. 

**Table 2:** Mean scores of sleep quality and its domains between and within the Aromatherapy (n=36) and Control (n=37) groups at the baseline and 4 weeks after the intervention

Variables	Study group	Stage of intervention		**P value (within groups)
		Before Mean±SD	After Mean±SD	
PSQI score	Citrus aurantium	12.08±2.1	5.75±1.33	<0.001
*P value (between groups)	almond oil	12.4±1.65	13.00±1.59	0.07
		0.46	<0.001	
Sleep mental quality	Citrus aurantium	0.69±0.57	0.22±0.42	<0.001
*P value (between groups)	almond oil	1.86±0.58	1.51±0.90	0.40
		0.22		<0.001
Sleep latency	Citrus aurantium	2.50±0.65	1.20±0.51	<0.001
*P value (between groups)	almond oil	2.62±0.49	2.50±.0.55	0.12
		0.63	<0.001	
Sleep duration	Citrus aurantium	2.11±1.08	0.80±1.66	<0.001
*P value (between groups)	almond oil	2.18±0.99	2.45±0.69	0.70
		0.65	<0.001	
Sleep efficiency	Citrus aurantium	1.05±0.71	0.55±0.60	<0.001
*P value (between groups)	almond oil	1.10±0.99	1.05±1.05	0.65
		0.22	<0.001	
Sleep disturbances	Citrus aurantium	2.22±0.42	1.41±0.50	<0.001
*P value (between groups)	almond oil	2.33±0.34	2.91±0.58	0.41
		0.28	<0.001	
Use of sleeping medication	Citrus aurantium	0.98±2.19	0.55±0.52	<0.001
*P value (between groups)	almond oil	1.16±0.95	1.20±0.93	0.92
		0.71	<0.001	
Daytime dysfunction	Citrus aurantium	2.22±0.42	1.41±0.60	<0.001
*P value (between groups)	almond oil	2.43±0.60	2.43±0.50	0.84
		0.68		<0.001

*Independent t-test; **Paired t-test

The mean difference between the baseline and post-test of PSQI score and those of its domains was calculated using ITT analysis method. ITT analysis was performed to determine the comparability of the baseline between the groups by randomization. The results of per protocol and ITT analyses showed no significant differences between the two groups (P=0.34 and P=0.51, respectively).

## DISCUSSION

This study examined the effect of aromatherapy using Citrus aurantium on sleep quality in postmenopausal women. After the intervention, the mean of PSQI global and component scores in the group who received Citrus aurantium was significantly lower than that of the control group. These results suggest that aromatherapy with Citrus aurantium improves the quality of sleep in postmenopausal women. 

Consistent with our results, a previous study compared the effects of lavender and bitter orange on sleep quality in postmenopausal women; it was shown that bitter orange and lavender significantly improved the mean sleep score compared with the control group. ^33^ It should be noted that the intervention was conducted orally for 8 weeks on 156 postmenopausal women in the mentioned study, while in our study aroma was used for 4 weeks on 80 postmenopausal women. Therefore, Citrus aurantium improves the sleep quality both when used orally and in aromatherapy; the mean score of sleep quality in this study was similar to that in the mentioned study (5.75±1.33 vs. 5.48±2.48, respectively), both of which obtained using Pittsburgh questionnaire. This result can be explained by the fact that the linalool in Citrus aurantium, which interacts with gamma-amino butyric acid receptors in the central nervous system, has a sedative action. ^34^

The results of another study showed that using the essence of 10% Citrus aurantium aroma, for three successive nights, improved the sleep quality in cardiac patients including those with ischemic heart disease, candidate for heart surgery and heart beat disorders, who were admitted to the cardiac care unit. ^35^ In another study conducted on 80 elderly patients with heart failure, aromatherapy with two drops of 10% citrus aurantium essential oil was performed on a cotton ball for twenty minutes for three consecutive nights; the sleep quality was improved without any side effects. ^36^ The results of both studies are in line with those of the present study; however, the participants in the present study did not suffer from any mental or physical diseases.

Anyway, citrus aurantium essential oil has shown the same improving effects on sleep quality in different populations; for example, in a study in which female nursing students received Citrus aurantium aromatherapy for 14 nights, the results showed significant improving effects on the students’ sleep and anxiety. In this study, the subjects inhaled 4-5 drops of the essence of Citrus aurantium for 15 min over 14 consecutive nights. The researchers suggested that aromatherapy with Citrus aurantium can be used as a useful method to improve sleep quality and reduce anxiety in students living in dormitories. ^37^ This result is consistent with those of the present study. However, in our study, the variable of anxiety was not measured.

However, in a research which assessed the effect of aromatherapy using lavender on sleep quality of nursing students, ^38^ the results showed that the lavender aromatherapy did not improve the sleep quality in students. In this study, the intervention was performed by dripping two drops of essential oil on a non-absorbent handkerchief for 7 nights and keeping it at a distance of 20 cm from the nose for 20 minutes. The discrepancy between the results of the present study and that of the mentioned study may result from differences in the method of intervention, number and type of study population, duration of drug administration, and medication type. 

In line with the results of present study, the researchers found that aromatherapy using bitter orange extract can improve the sleep quality in diabetic patients. In this study, patients in the experimental group inhaled 8 drops of bitter orange extract 20% at three consecutive nights, and the data collection tools was Richard Campbell sleep questionnaire. According to their results, the mean of total sleep quality score in the experimental group was significantly higher than that of the control group. ^23^ This result is consistent with the findings of the present study; despite the differences in the study population, type of questionnaires used, and differences in the type and manner of drug administration, both drugs improved the women’s sleep quality. In the present study, women in the intervention group inhaled 2 drops of essential oil of Citrus aurantium, twice a day for 4 consecutive days in a week, and continued it for 4 weeks.

Regarding the effect of aromatherapy with different drugs on sleep quality, it is believed that the aroma activates the olfactory nerve and subsequently stimulates the limbic system. Depending on the type of aroma, the neuron cells release different neurotransmitters including ankylpholine, endorphin, noradrenaline, and serotonin. On the other hand, due to the association of the sense of smell with other human senses and mind, aromas can exert their effects both mentally and physically. In fact, the odor can change human’s feelings. ^39^

The first limitation of this study was that we did not assess other factors that could contribute to sleep quality (e.g., the dietary pattern of the participants, stress, etc.), nor did we check the onset of sleep disorder. Furthermore, we studied only the short-term effects of aromatherapy. On the other hand, this study was the first to assess the effect of Citrus aurantium aroma on the quality of sleep in postmenopausal women. Also, the random allocation of subjects to avoid selection bias and the use of blinding method to reduce the risk of bias during data collection were the other strengths of the study.

## CONCLUSION

According to the findings of this study, it appears that aromatherapy using Citrus aurantium is useful in the treatment of sleep disorders in postmenopausal women. Health care providers in in-service training classes should be aware of the benefits of Citrus aurantium in reduction of sleep disorders, so that they can recommend its use while counseling postmenopausal women. Therefore, informing postmenopausal women and advising them to use the Citrus aurantium aroma for reduction of sleep disorders are suggested. However, further clinical trials with longer follow-up periods and on participants with physical diseases are needed to confirm these findings. 
